# Detection of a chikungunya outbreak in Central Italy, August to September 2017

**DOI:** 10.2807/1560-7917.ES.2017.22.39.17-00646

**Published:** 2017-09-28

**Authors:** Giulietta Venturi, Marco Di Luca, Claudia Fortuna, Maria Elena Remoli, Flavia Riccardo, Francesco Severini, Luciano Toma, Martina Del Manso, Eleonora Benedetti, Maria Grazia Caporali, Antonello Amendola, Cristiano Fiorentini, Claudio De Liberato, Roberto Giammattei, Roberto Romi, Patrizio Pezzotti, Giovanni Rezza, Caterina Rizzo

**Affiliations:** 1 Department of Infectious Diseases, Istituto Superiore di Sanità, Rome, Italy; 2 These authors contributed equally to this article and share first authorship; 3 Istituto Zooprofilattico Sperimentale Lazio e Toscana, Rome, Italy; 4 Local Health Authority Roma 6, Albano, Italy

**Keywords:** Chikungunya, Italy, autochthonous cases, invasive mosquitoes

## Abstract

An autochthonous chikungunya outbreak is ongoing near Anzio, a coastal town in the province of Rome. The virus isolated from one patient and mosquitoes lacks the A226V mutation and belongs to an East Central South African strain. As of 20 September, 86 cases are laboratory-confirmed. The outbreak proximity to the capital, its late summer occurrence, and diagnostic delays, are favouring transmission. Vector control, enhanced surveillance and restricted blood donations are being implemented in affected areas.

## Outbreak identification and investigation

On 6 and 7 September 2017, the National reference Laboratory for arboviral infections based at the National Institute of Health, Italy, received serum and urine samples from three patients with a history of high fever (>38°C), severe joint pain and an itching skin rash. Symptoms had started while they were on holiday near the coastal town of Anzio, in the province of Rome, Lazio region (ca 58 km from Rome). The dates of symptom onset were 5 and 11 August and 2 September respectively. The patients lived in the same home, and none had travelled to chikungunya, dengue or Zika endemic areas in the two weeks before symptom onset. The two patients who developed symptoms at the beginning of August were chikungunya IgM positive and the infection was confirmed through a neutralisation test (PRNT). The third patient, who was symptomatic at the time of sample collection, was IgM positive and PRNT borderline (PRNT50 ≥ 1:10). Chikungunya virus was detected by RT-PCR followed by nested PCR in both serum and urine and was isolated from urine. 

The regional health authorities immediately implemented measures around this initial cluster, which are described in the national plan for chikungunya surveillance and control [1]. The three patients had referred to other individuals in their neighbourhood who had developed similar symptoms since the beginning of August. Epidemiological surveillance was enhanced to identify additional cases (case finding trough general practitioners (GPs), paediatricians, emergency rooms and hospitals). As of 20 September 2017, 86 confirmed autochthonous cases of chikungunya were reported to the regional surveillance system; the investigation is ongoing. These were found among people living in Anzio, or people who had travelled there 15 days before symptom onset. Cases from Rome were also identified, with either travel history to Anzio or to other endemic areas [2]. The primary viraemic case who imported the virus into the region has not been identified so far.

## Entomological investigation

On 7 and 8 September 2017, adult mosquitoes were collected in the Anzio municipality by using BG Sentinel traps baited with BG-Lure (Biogents, Regensburg, Germany) and captured directly using handheld electric aspirators on vegetation or on exposed skin. Larval collections were performed using dippers or droppers by inspecting artificial containers (removable or non-removable), as potential larval breeding sites. Adults and larvae of *Ae. albopictus* (tiger mosquito) and *Culex pipiens* were found in the Anzio municipality area. Mosquito adults were sorted into 12 pools to determine the species, distinguish males from females and to test for chikungunya virus. The number of specimens in each pool ranged from 1 to 13. Among 53 mosquitoes, one pool of 12 *Ae. albopictus* females which had been collected near the house of the first three cases identified in Anzio was found to be chikungunya virus positive by PCR. The virus was isolated.

## Phylogenetic analysis of patient and mosquito sequences 

Sequences of the PCR amplicons of the virus envelope (E)1 gene from the patient (GenBank numbers: LT908477 and LT908478) and from the mosquitoes were identical (GenBank number: LT908476), and also showed a 100% similarity with the sequence of a chikungunya East Central South African (ECSA) strain involved in an ongoing epidemic in Pakistan [3,4], which does not carry the A226V mutation. In a phylogenetic analysis, the Italian sequences also clustered with ESCA strain sequences obtained from different parts of the world (Figure).

**Figure:**
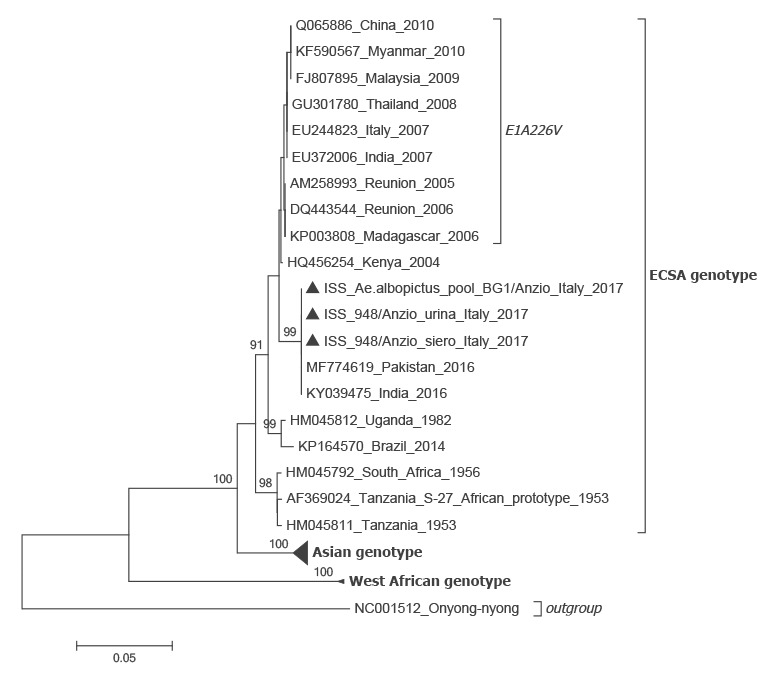
Neighbour-joining phylogenetic analysis of sequences derived from chikungunya virus positive samples obtained in this study, Italy, August−September 2017

ESCA: East Central South African.

The phylogenetic tree was constructed using Tamura-Nei model with 1,000 bootstrap reiterations. For each sequence in the tree, GenBank accession number/country of origin of the infection/year of the infection are reported. Sequences characterised in this study are indicated by a black triangle (for the patient samples ‘siero’ indicates a sequence derived from a serum sample (GenBank number: LT908478), and ‘urina’, a sequence from a urine sample (LT908477); the GenBank number for the sequence from mosquitoes is LT908476). Sequence amplicons were obtained using method reported in Edwards C. et al., J Clin Virol. 2007 [11]. The scale bar indicates the percentage of diversity. Bootstrap values over 90% obtained from 1,000 replicate trees are shown for key nodes.

Two clusters are highlighted in the Figure. The first, identifiable as ‘ESCA genotype’, indicates sequences of the ESCA genotype. The second cluster, identifiable as ‘E1 A226V’, includes all sequences with the A226V mutation in the envelope (E)1 gene, a mutation, which is involved in the increased susceptibility of *Aedes albopictus* for infection and transmission of chikungunya virus.

## Control measures

On 8 September Italy launched a first alert through the Early Warning Response System (EWRS) in order to alert other European Union countries about the ongoing outbreak. In the affected municipality, vector control activities against larvae and adult mosquitoes were performed and the population was advised to take measures to avoid mosquito bites and to remove all potential breeding sites from their gardens. Moreover, measures such as clinical evaluation of blood donors, gathering post-donation information for donors residing in the Lazio region, and application of 28 days deferral of donors who visited the municipality of Anzio since 1 August 2017 were reinforced [5].

## Background

Chikungunya is a self-limiting mosquito-borne viral disease characterised by arthralgia/arthritis primarily of the wrists, knees, ankles and small joints of the extremities lasting from days to months. Between one third and half of the patients develop a generally non-pruritic maculopapular rash primarily of the trunk and limbs 1–12 days after the onset of arthritis that resolves in 7–10 days. Myalgia, fatigue, fever and lymphadenopathy are common [6]. Complications are rare; however, Chikungunya leads to (self-perceived) long-term sequelae in a considerable proportion of patients [7]. The incubation period ranges from 1 to 10 days (average 3 days) [8].

The aetiological agent is a single strand, positive sense RNA virus of the Alphavirus genus, Togaviridae family of which three main genotypes have been identified: Asian, West African and ECSA [8]. The virus is transmitted by mosquitoes of the *Aedes* spp. primarily *Ae. aegypti* and *Ae. albopictus*. Transmission through transfusion/transplantation has been demonstrated in animal models. 

Chikungunya is endemic in Africa, south-east Asia, the Indian subcontinent, the Pacific region and in tropical regions of the Americas (since 2013). In Europe, autochthonous cases, which were linked to imported cases, were detected in France in the Var department, in September 2010 and in the city of Montpellier, Hérault department, in October 2014. In August 2017, a cluster of locally acquired cases consisting of four confirmed and one probable case was also described in the Var department [8]. 

## Discussion

The outbreak described here is the second autochthonous chikungunya outbreak detected in Italy. The first outbreak, with more than 200 reported cases, occurred between July and September 2007 in the north east of the country, near the Adriatic coast, in the province of Ravenna [9]. Subsequent to it, Italy set up a national plan for the surveillance and control of the disease [1]. The aim of this surveillance system is to monitor imported cases and local transmission, identify outbreaks in a timely fashion, and to prevent transmission from substances of human origin (SoHO). The surveillance is enhanced during the high vector activity season (1 June–31 October). The competent vector, *Ae. albopictus*, which was first detected in north-western Italy in 1990 [9], is now widely established throughout the country, including the Lazio region. Overall between 2014 and 2016, a total of 128 possible/probable/confirmed imported cases of chikungunya have been notified to the Italian health authorities (mean: 43 cases/year; range: 25 cases in 2015 and 70 cases in 2014). Most cases had a travel history to Central and South America [1].

Anzio, one of the locations where autochonous chikungunya cases were currently detected, is a holiday resort less than one hour drive away from Rome. Many families visit the area until the beginning of the school year, which in 2017 was on 14 September. During the summer, the population density increases in the area owing to the easy commute from Rome. Viraemic individuals exposed in August 2017 are likely to have travelled around resulting in new introductions in receptive areas and challenging the implementation of control measures. The delayed identification of the autochthonous cases in Anzio, a coastal recreational area close to the capital towards the end of summer may have triggered the local transmission in Rome. This suggests an extension of the outbreak and the presence of secondary multifocal transmission chains [10].

The lack of access to closed summer houses after mid-September may hamper vector control activities. Summer 2017 has been characterised by particularly high temperatures and by an unusual dry period from May to August, factors that would normally prevent high mosquito density. However, other concomitant factors favoured the activity and abundance of the tiger mosquito including the presence of numerous peridomestic ‘man-made’ breeding sites within constantly irrigated private gardens.

The identification of the viral strain involved in the current Italian outbreak as one very close to the ECSA strain involved in the ongoing epidemic in Pakistan is not surprising, even if this virus it is not carrying the A226V mutation, which has been involved in the increased susceptibility of *Ae. albopictus* for infection and transmission of chikungunya virus. Other yet undefined mutations might be responsible for the vector competence of *Ae. albopictus*, allowing it to sustain the present outbreak. The fact that we found chikungunya RNA in a small size mosquito pool, suggests a high level of virus circulation in the area. The investigation is still ongoing and, due to enhanced surveillance and testing, further cases have been identified, with as of 22 September, 102 laboratory-confirmed cases.
